# Machine Learning-Driven Sensitivity Analysis for a 2-Layer Printed Circuit Board Inductive Motor Position Sensor

**DOI:** 10.3390/s26030879

**Published:** 2026-01-29

**Authors:** Qinghua Lin, Devin Sullivan, Douglas Moore, Donald Tong

**Affiliations:** 1 CTS Corporation, 905 W Blvd. North, Elkhart, IN 46514, USA; doug.moore@ctscorp.com (D.M.); donald.tong@ctscorp.com (D.T.); 2 Independent Researcher, Malden, MA 02148, USA

**Keywords:** inductive motor position sensor, routing strategy, machine learning, extreme gradient boosting, SHapley Additive exPlanations, Fourier series, accuracy

## Abstract

Motor position sensors are critical parts for traction motors control in electrified automotive powertrains. As motors are becoming more compact due to the advance of technology the packaging space for motor position sensors is becoming increasingly restricted. This study presents a two-layer (2L) printed circuit board (PCB) routing strategy for inductive motor position sensors with limited area. A prototype was fabricated and tested on a test bench using a comprehensive design of experiments that contains 625 combinations of X- and Y-offsets, tilt angle, and airgap at various levels (±0.5 mm in X/Y, ±0.5° tilt, 1.9–3.1 mm airgap). Across the tolerance box, the accuracy under all test cases remained within ±1 electrical degree. The accuracy analysis through Fourier series on a circle shows that the DC offset and magnitude mismatches of the 3 Rx signals are the dominant error contributors due to the routing modification. An Extreme Gradient Boosting (XGBoost) model was trained and validated with R^2^ = 0.9951. A comparison with a Multiple Linear Regression baseline (R^2^ = 0.0565) demonstrates that installation-induced accuracy degradation is inherently non-linear. The SHapley Additive exPlanations (SHAP) and interaction intensity analysis identified tilt and Y-offset as dominant error drivers, revealing a strong coupled influence (interaction intensity = 0.9581). The model revealed a mild *Y*-axis asymmetry introduced by routing modifications. This integrated workflow provides a general, quantitative framework for optimizing and analyzing inductive sensor layouts and establishing installation tolerances.

## 1. Introduction

The global move toward electrified mobility [1] requires precise control on the performance and reliability of electric motor control systems. The demand for higher torque density and lower torque ripple is driving the development of increasingly complex traction motor topologies and harmonic-field engineering approaches—such as PM-assisted synchronous reluctance motors with advanced magnet configurations [2], flux-modulated PM machines whose airgap-harmonic content must be systematically characterized [3], and PM vernier machines where torque ripple suppression is explicitly addressed from time- and space-harmonic perspectives [4]. Accurate rotor position feedback is essential for maximizing the efficiency and torque output of traction motors [5,6,7]. Motor position sensors are critical to achieving this task, which must survive the severe operational environment of the motor, including high temperatures, intense vibration, corrosive contaminants, and significant electromagnetic interference (EMI) [8]. Traditionally, electromechanical resolvers have been the established standard for high-accuracy and robust position sensing in traction motor applications [8,9,10]. Resolvers are reliable and robust, but they come with drawbacks: their wound-coil stator and rotor structures contribute to significant weight and bulk, complexity in manufacturing, and higher unit cost due to precision winding requirements. While excellent in performance, their form factor and cost implications are increasingly challenging for high-volume, compact EV powertrains. Other encoder technologies, such as optical or magnetic encoders, often face their own limitations in harsh automotive environments, including sensitivity to dirt, condensation, stray magnetic fields, and temperature drift.

Inductive motor position sensors offer an alternative. Utilizing printed circuit board (PCB)-based coil structures reduces weight and size, lowers manufacturing complexity, and exhibits resistance to stray fields. This makes them ideal for the constrained and demanding environment of modern traction motors [11]. In practice, inductive position sensing is commonly realized with PCB-integrated coils, since coil dimensions on the order of tens of millimeters are typically required to generate sufficient signal strength and accuracy within compact sensor housings [12]. Inductive position sensor devices operate by driving a high-frequency excitation current through one or more transmit (Tx) coils, generating an alternating magnetic field. A conductive target modulates this field through eddy-current induction. The resulting mutual coupling between the Tx coils and a set of receive (Rx) coils varies as a function of the rotor angle. By geometrically encoding the Rx coils (often sin/cos or multiphase patterns), the induced voltages reconstruct the absolute rotor position through arctangent demodulation or resolver-like signal processing [13,14]. In an ideal design, the Tx coil generates a spatially uniform magnetic field, and the Rx coils maintain perfect spatial sensitivity, ensuring high linearity and immunity to misalignment. Any disruption of the underlying coil symmetry directly impacts accuracy [15,16]. This sensitivity is particularly pronounced in 360° architectures, where off-axis shift and tilt can distort the eddy-current distribution and produce periodic angle errors, while airgap variations primarily modulate coupling strength and signal-to-noise ratio; therefore, installation tolerances and coil layout symmetry must be considered jointly when pursuing high accuracy in practical assemblies [12,17].

For high-precision inductive MPS, a 4-layer (4L) PCB architecture supports symmetric Tx/Rx routing, well-controlled return paths, and minimal disturbance to the ideal magnetic field distribution. However, industry pressure toward cost reduction and miniaturization increasingly motivates the transition to 2-layer (2L) PCBs. In many practical applications the Tx coil must span both layers to satisfy dimensional requirements. As a result, routing all Tx and Rx terminals back to the integrated circuit (IC) becomes challenging. These geometric modifications break the magnetic symmetry guaranteed by a 4L stack-up and inevitably introduce excitation-field distortion, which manifests as increased non-linearity in the accuracy. Prior PCB-focused inductive sensing studies further indicate that layer allocation and routing constraints can create systematic asymmetry—for example, placing Rx coils on different PCB sides can reduce via usage but introduces unequal target distance and channel amplitude imbalance, which can significantly worsen linearity in non-optimized implementations—highlighting that PCB stack-up decisions are not merely manufacturing choices but directly shape the electromagnetic symmetry seen by the target [17]. Therefore, the core engineering question is how to achieve a minimally distorted coil topology. In addition, validating the robustness of such asymmetric designs is challenging because routing-induced geometric distortion can interact non-linearly with mechanical installation tolerances, making the resulting error mechanisms difficult to quantify using traditional linear sensitivity analyses.

Machine learning (ML) methods are being integrated across the sensors’ lifecycle—from design and optimization to signal interpretation and deployment monitoring—and it provides a general framework for improving sensor accuracy and robustness under practical operating conditions [18]. In references [19,20,21], they applied ML in the calibration and evaluation of sensors, offering a superior alternative to traditional linear calibration techniques and enhancing the accuracy of low-cost sensors. Payette et al. employed a deep neural network (DNN) architecture to perform regression analysis on data from a low-cost, low-accuracy array, effectively identifying hidden patterns to enhance measurement precision [22]. To demonstrate that traditional linear models cannot sufficiently capture the non-linear dependencies in our specific architecture, we utilize a Multiple Linear Regression model as a baseline for comparison. At the same time, engineering adoption increasingly demands interpretability: explainable methods such as SHAP are widely used to overcome “black-box” limitations by quantifying how each input variable contributes to the prediction (including the directionality of its influence), enabling actionable insight rather than purely predictive accuracy [23]. This direction aligns with the broader movement toward physics-informed sensing, where ML is paired with physically meaningful predictors and feature-importance analysis to maintain consistency with known mechanisms and improve trust in model-guided decisions [24]. Notably, recent eXplainable Artificial Intelligence (XAI) surveys report that SHAP is most frequently paired with tree-ensemble models in quantitative tasks where both predictive performance and interpretability are paramount [25]. Motivated by these developments, we employ Extreme Gradient Boosting (XGBoost)—which is powerful in modeling complicated interactions within high-dimensional datasets [26]. By integrating the XGBoost model with SHapley Additive exPlanations (SHAP) [27,28], we gain insight into the underlying relationships between installation tolerances and sensor accuracy.

This work establishes that a cost-driven two-layer (2L) PCB implementation can achieve high-precision inductive motor position sensing when electromagnetic symmetry is treated as an explicit design objective under routing constraints. Our primary contribution is a 2L Tx/Rx coil topology that preserves effective symmetry within a strict footprint while accommodating unavoidable terminal-routing modifications. We validate this framework experimentally using a prototype evaluated over a 625-condition tolerance box spanning misalignment and airgap variations. Across this full tolerance space, the 2L layout maintains ±1 electrical degree accuracy, establishing a quantitative performance baseline for 2L routing under realistic installation variability. Beyond reporting sensor accuracy, we introduce a physical-to-digital mapping workflow in which the measured angle error is Fourier-decomposed into harmonic components. By isolating these harmonics, we can directly correlate periodic error patterns to physical layout asymmetries, effectively turning raw sensor data into actionable design feedback. By further integrating XGBoost with SHAP, we quantify the non-linear interaction effects of installation tolerances on these specific error components. This provides a data-driven foundation for tolerance allocation and targeted design refinement, bridging the gap between physical PCB layout constraints and robust sensor performance.

## 2. Materials and Methods

### 2.1. 2L-PCB Inductive MPS Design

**Figure 1:**
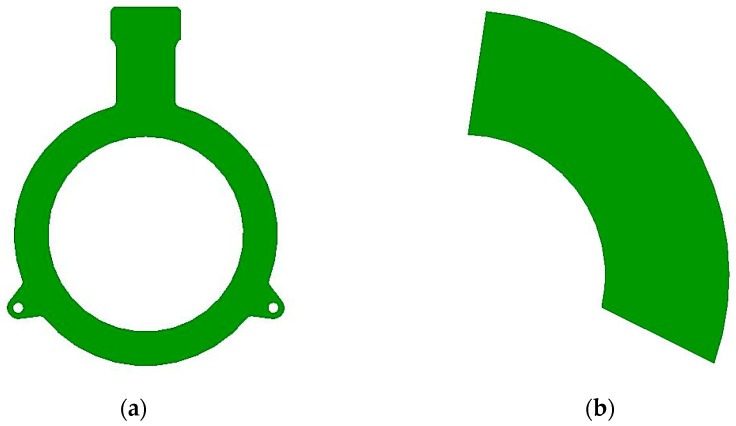
Inductive MPSs configurations: (**a**) full-circle configuration; (**b**) partial-circle configuration.

**Figure 2:**
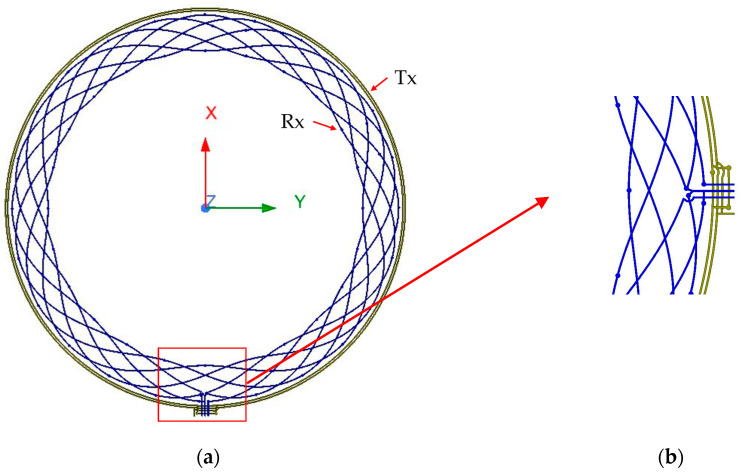
L configuration. (**a**) Layout of the Tx/Rx coils; (**b**) zoomed-in Tx/Rx routing to the IC.

Inductive MPSs generally have either a full-circle or partial-circle shape. Full-circle designs, as shown in Figure 1a, allow circular Tx coils to generate a uniform magnetic field along the Rx coils shown in Figure 2. These configurations have strong signal strength and high robustness against installation variations. Partial-circle designs, illustrated in Figure 1b, implement only a portion of the full circle. The partial-circle design enables compact layout and low cost but disrupts field symmetry and very susceptibility to misalignment and mechanical tolerances. The present work focuses on full-circle configurations.

There are mainly three cases regarding PCB layers and arrangement of Tx/Rx coils when balancing performance, manufacturability, and cost (here, we are not considering any shielding layers). The selection between a four-layer and two-layer configuration depends primarily on available area for coils layout, Rx coils’ signal strength, and the degree of routing distortion that can be tolerated without compromising signal integrity.

Case 1—Four-layer (4L) PCB: In space-constrained applications, the 4L configuration provides two main advantages: (1) it preserves Tx/Rx magnetic-field symmetry to maintain strong, balanced Rx signals, and (2) it offers the routing flexibility needed to connect the coils to the IC. These benefits make the 4L option a robust choice when PCB area is limited.

Case 2—Two-layer (2L) PCB with sufficient area: When adequate PCB area is available, the Tx coils can be routed entirely on a single layer. This allows Tx and Rx traces to connect to the IC without modifications. This layout provides excellent field balance and accuracy, as well as the most cost-efficient configuration.

Case 3—Two-layer (2L) PCB with limited area: In this application where PCB area is restricted but diameter is bigger than case 1, a 2L configuration often becomes unavoidable for cost reasons. If the Tx occupies a single layer like the previous case, the remaining area for Rx routing may be insufficient to induce a strong voltage in Rx. As a result, the Tx and Rx coils need to be laid out in both layers. This will introduce asymmetries that increase non-linearity due to the modification of coils routing shown in Figure 2. This configuration, though challenging, represents a realistic case for mass-produced automotive sensors and forms the focus of this study.

Figure 2a shows the full layout of the concentric Tx/Rx coils, while Figure 2b zooms in the routing region. The zoomed view highlights the unavoidable geometric asymmetry induced when directing the Tx and Rx traces toward the interface IC. This modification is the primary source of the magnetic field imbalance in compact 2L designs. The guiding principle for such modifications is to ensure minimum distortion of the Tx/Rx coil layout while strictly adhering to manufacturing clearance requirements between traces. The objective of this work is to experimentally evaluate the impact of this routing modification and demonstrate that, despite being unavoidable in compact 2L architectures, the sensor can still achieve the required accuracy and robustness.

The excitation frequency of the inductive sensing system is constrained by the automotive-grade sensing ASIC, which requires synchronous demodulation within a 2–5 MHz band [29]. To ensure robust operation, the Tx resonant frequency is designed at 3.5 MHz. This selection provides sufficient margin against frequency shifts induced by temperature-dependent capacitance variations and component tolerances. Consequently, sensitivity analysis focused on amplitude management and geometric scaling rather than frequency variation. While increasing the Rx coil area can raise the induced signal and improve SNR, the ASIC imposes an approximately 100 mV limit. Exceeding this threshold causes saturation and clipping that corrupt the demodulated output. Finally, although pole-pair selection is typically dictated by the customer’s application and is therefore not treated as an optimization variable in this study, it remains a critical factor in performance. For a given electrical resolution, increasing the pole-pair count improves the effective mechanical-angle resolution, though it can also increase high-order harmonics in the Rx signal and potentially introducing non-linearity.

Magnetic asymmetries caused by mechanical installation tolerances (tilt, misalignment, and airgap) manifest as DC offsets. In reference [29], it indicates that thermal drift is not an independent random variable but is directly proportional to the magnitude of the initial offset that must be compensated. If a design achieves a very low offset at room temperature through an optimized two-layer (2L) routing strategy, the subsequent thermal drift error remains minimal across the standard automotive operating range of −40 °C to 150 °C.

**Figure 3:**
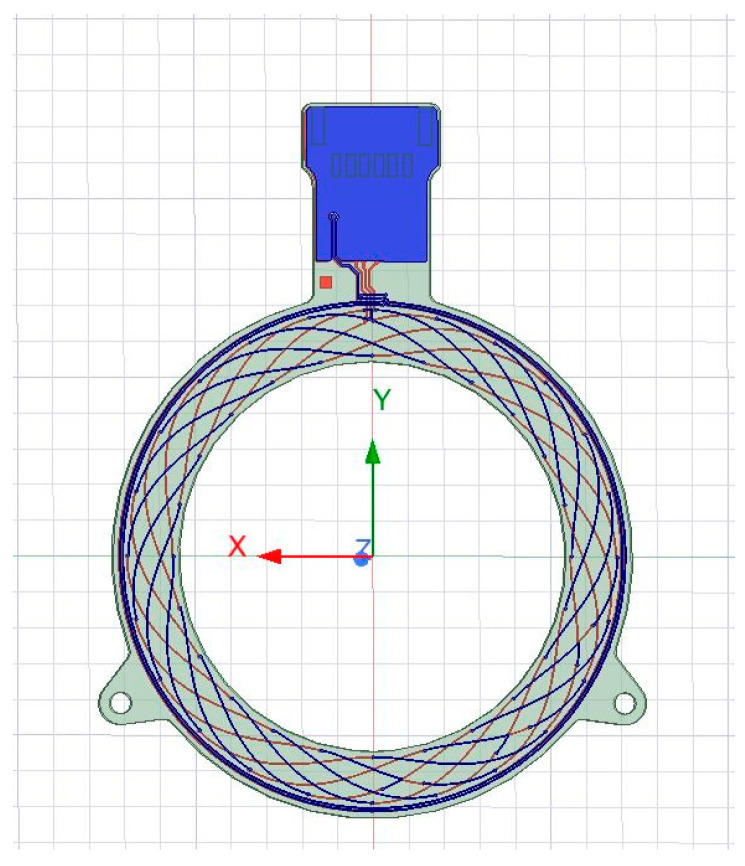
Proposed 2L PCB coil layout.

The two-layer prototype, shown in Figure 3, evaluated in this work has an outer diameter of 73.2 mm and an inner diameter of 54.5 mm. The Tx coils consist of four turns with two turns on each layer. Both Tx and Rx coils use a trace width of 0.15 mm and 35 µm copper thickness. The excitation frequency was designed at about 3.5 MHz.

### 2.2. Design of Experiments

**Figure 4:**
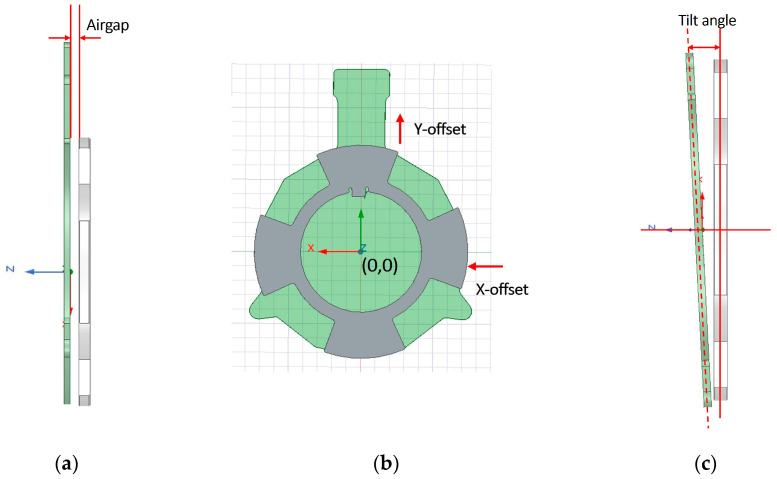
Installation definition: (**a**) airgap; (**b**) X, Y offsets and the origin; (**c**) tilt angle along Y direction.

A structured experimental design was implemented to quantify how mechanical installation or life-long deviations affect the accuracy and robustness of the two-layer (2L) inductive MPS. The mechanical factors—airgap, misalignment along the X- and Y-axes, and tilt angle—were considered within realistic application limits. Figure 4 illustrates the definition of each installation variable: (a) the airgap between the sensor PCB and the rotating target, (b) X and Y offset, and (c) the tilt angle (θ) representing tilted PCB compared to target along the *Y*-axis. The nominal condition is defined at x = 0 mm, y = 0 mm, airgap = 2.5 mm, and θ = 0°.

While signal delays influence high-speed precision, the sensing ASIC has a dedicated DSP Phase Tracking unit that calculates the instant angle and automatically compensates for processing delays using real-time speed and acceleration estimates [29]. Consequently, the sensor’s accuracy is effectively independent of rotational speed within the specified automotive operational envelope. This is consistent with our in-house test result of inductive MPS that accuracy is independent of rotational speed within the specified automotive operational range. Thus, all tests were conducted at a fixed speed of 2000 RPM in this study. In addition, this speed lies near the mid-range of our onsite servo drive capability (maximum 5000 rpm) and was chosen to ensure a consistent condition for all tests. Each factor was evaluated at five levels spanning the normal mechanical tolerance range. The resulting full-factorial matrix, summarized in Table 1, covered 625 combinations of airgap, X/Y offset, and tilt. This factorial structure enables quantification of both single-factor and interaction effects while keeping the total test count within manageable limits.

For each condition, data recording began after the motor speed reached the target and stable. During each test, the sensor’s sine and cosine differential signals were captured simultaneously with the encoder reference angle.

### 2.3. Experimental Setup

**Figure 5:**
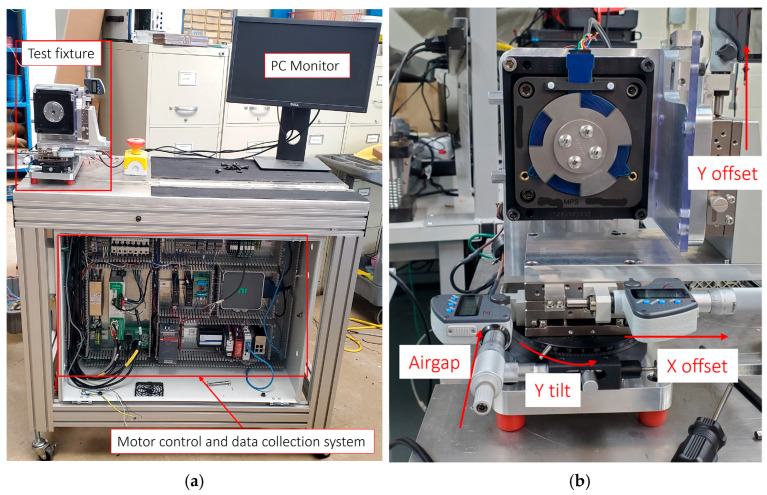
Experimental setup: (**a**) whole test cart; (**b**) fixture for sensor and motor.

The experimental setup is illustrated in Figure 5. The system was capable to precisely impose and measure geometric misalignments—including airgap, lateral offsets, and tilt angle—while recording synchronized sensor and reference signals. The test cart [Figure 5a] includes the test fixture, PC monitor, and data acquisition and motor control system. The test fixture [Figure 5b] allowed the sensor to be mounted coaxially with the rotating metallic target. The target was driven by a servo motor. The airgap between the sensor and target was adjustable by means of a micrometer-controlled *Z*-axis stage. Lateral X and Y offsets were introduced using precision translation stages with 0.001 mm resolution, while angular tilt was applied through a rotary stage with accuracy of 0.01°.

Sensor signals were recorded using a National Instruments PCIe-6361 card with simultaneous analog sampling at 25 KHz per channel. The reference mechanical angle was obtained from a high-resolution optical encoder (18-bit) mounted on the same shaft. Both sensor and encoder signals were captured synchronously using a shared hardware trigger. Before the experiments, analog channel-to-channel phase skew in the DAQ system was characterized by applying the same input signal to all recording channels. The resulting time offset was measured and compensated in data collection system to ensure precise alignment between the sine and cosine channels. Accurate channel synchronization is particularly critical for high-speed motor applications, where even sub-microsecond skew can introduce a significant phase shift between sine and cosine waveforms, distorting the calculated electrical angle and leading to incorrect evaluation of sensor performance [6,30,31]. All measurements were performed at room temperature (23 ± 2 °C). For each test condition 5 s length of data was captured.

### 2.4. Methods

#### 2.4.1. Definition of Sensor Accuracy and Target Variable

The accuracy of the sensor was quantified as the deviation between the electrical angle calculated from the MPS signals and the reference electrical angle obtained from the encoder, as expressed in Equation (1).

(1) $${∆\theta }_{i}=\theta_{mps,i}-\theta_{ref,i}$$

The MPS electrical angle was computed from the sine and cosine signals:

(2) $$\theta_{mps,i}\;=\;atan(\frac{{sin}_{i}}{{cos}_{i}})$$

The reference electrical angle was derived from the encoder’s mechanical angle according to

(3) $$\theta_{ref,i}=mod(\varphi_{i}\times N+\theta_{offset},\;360)$$

where $\theta_{mps,i}$ represents calculated electrical angle from inductive MPS, which is calculated by Equation (2); $\theta_{ref,i}$ is the reference electrical angle converted from encoder angle, which is a mechanical angle. The relationship between electrical angle and mechanical angle can be expressed in Equation (3), where $\varphi_{i}$ represents the encoder reading; N is the number of pole-pairs of the motor; $\theta_{offset}$ is a fixed electrical angle offset determined under nominal mechanical alignment. The ${sin}_{i}$ and ${cos}_{i}$ are the inductive MPS voltage outputs. For each sensor prototype, the offset was first determined under the nominal alignment condition and subsequently held during accuracy evaluations at all conditions.

The voltage from Rx coils (U, V, and W) can be expressed as Equation (4). $A_{u,i}$, $A_{v,i}$, and $A_{w,i}$ are the harmonic coefficients of the 3 Rx signals, representing the signal magnitudes. Ideally, all the even harmonics are rejected by the clockwise and counterclockwise turning loops design in the coils, while odd terms are left in the system [13]. $c_{u}$, $c_{v}$, and $c_{w}$ are the DC offsets. These DC offsets are caused by imbalance between the clockwise and counterclockwise winding in the Rx coils [15]. In a perfect design and manufacturing process, we expect U, V, and W have identical harmonic coefficients without any DC offset because any difference among the harmonic coefficients and existence of DC offset will all turn into angle errors.

(4) $$\left\{\begin{array}{c} U=\sum_{i=1}^{\infty }A_{u,i}cos\left(i\theta \right)+c_{u}\\ V=\sum_{i=1}^{\infty }A_{v,i}cos\left(i\theta -i\frac{2\pi }{3}\right)+c_{v}\\ W=\sum_{i=1}^{\infty }A_{w,i}cos\left(i\theta +i\frac{2\pi }{3}\right)+c_{w} \end{array}\right.$$

Since the angle error ∆θ is a periodic function of the electrical angle, it can be approximated by a Fourier series on the circle, as Equation (5). $A_{n}$ is the amplitude and $\varphi_{n}$ is the phase of nth-order harmonic. According to references [13,15], DC offsets in U, V, and W will show up in the angle error as first-order harmonic; mismatch of Rx signals amplitudes will express as second-order harmonic in the error; other higher-order harmonics in U, V, and W will enter the angle error as higher-order (>2 order) harmonics. By decomposing angle errors in Fourier fashion, we can identity the underlying design non-idealities.

(5) $$∆\theta =\sum_{n=1}^{n}A_{n}cos\left(n\theta +\varphi_{n}\right)+a_{0}$$

In the machine learning regression model, a target variable (y) is constructed as the model output expressed in Equation (6). For each mechanical condition, instead of directly using the sensor average error as the target, we normalized errors by comparing to the nominal condition average error and scaled by 100. Multiplying by 100 converts the normalized deviation into a bigger level, which improves numerical stability during model training and ensures that small variations in accuracy are adequately represented by the regression algorithm [32].

(6) $$y=\left[\frac{\sum_{1}^{n}\left(\left|\theta_{mps,i}-\theta_{ref,i}\right|\right)}{n}-{\left(\frac{\sum_{1}^{n}\left(\left|\theta_{mps,i}-\theta_{ref,i}\right|\right)}{n}\right)}_{nominal}\right]\times 100$$

**Figure 6:**
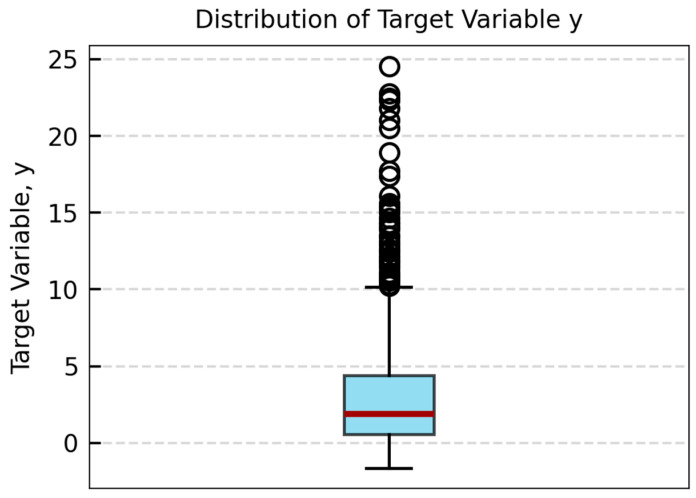
Target values for all the data, including training and validating datasets.

Figure 6 displays the distribution of all target variables. The boxplot highlights the spread of installation-induced accuracy deviations. This skewed distribution indicates that certain installation perturbations induce larger angle deviations than others. Characterizing the target variables in these areas provides insight for specific installation conditions that lead to elevated accuracy degradation.

#### 2.4.2. Extreme Gradient Boosting (XGBoost)

XGBoost is an ensemble learning technique based on gradient boosting decision trees, originally proposed by Chen and Guestrin [26]. It constructs an additive series of weak learners—typically shallow trees—where each subsequent tree is trained to correct the residual errors of the previous ensemble [33]. This sequential approach allows XGBoost to reduce both bias and variance, achieving strong generalization even with highly non-linear feature interactions. Its robustness, scalability, and high predictive performance on tabular datasets make it a great fit to model the non-linear relationship between mechanical installation tolerance and accuracy.

In this study, we implement the XGBRegressor from the xgboost 3.0.5 library in Python 3.10. Input features include X- and Y-offsets, airgap, and tilt angle. The model was trained with 70% of the dataset (437 cases) and validated on the remaining 30% (188 cases). To ensure robustness, a 5-fold cross-validation was conducted on the training set within the Optuna Bayesian optimization framework. The optimized hyperparameters included the number of estimators, learning rate, maximum tree depth, minimum child weight, subsampling ratio, column sampling ratio, L1 and L2 regularization terms, and the minimum loss reduction parameter. The model training process followed three stages:

1. Cross-validation optimization: For each Optuna trial, a 5-fold cross-validation was performed to compute the mean RMSE, which served as the objective metric to minimize.
2. Model selection and retraining: After 6000 trials, the best hyperparameters were identified and used to retrain the final model on the full training dataset.
3. Final evaluation: The optimized model was then applied to the independent 30% test dataset to generate predicted accuracy values.

#### 2.4.3. Baseline Model: Multiple Linear Regression (MLR)

In addition to XGBoost, a Multiple Linear Regression (MLR) model was implemented as a baseline to evaluate the necessity of capturing non-linear interactions between installation tolerance. To ensure a rigorous comparison, both models utilized the same data split and were subjected to identical 5-fold cross-validation protocols. Traditional linear models are widely used for sensor calibration but often struggle with the coupled, non-linear dependencies found in complex sensor architectures. The relationship is modeled as

$$y=k_{0}+\sum_{i=1}^{4}k_{i}x_{i}$$

where y is the target variable; $x_{i}$ represents the installation tolerances; $k_{i}$ are the regression coefficients. The performance of XGBoost and MLR models were assessed using the coefficient of determination (R^2^), root mean square error (RMSE), and mean absolute error (MAE) on the test dataset. A scatter plot of predicted versus measured accuracy values was also produced to visualize agreement between model predictions and true values.

#### 2.4.4. SHAP

To interpret the internal decision process of the XGBoost model and quantify the contribution of each geometric variable to the predicted sensor accuracy, the SHAP framework was used. SHAP provides a unified, theoretically grounded approach for assigning feature importance in complex machine learning models [27,28]. In the SHAP framework, each feature is treated as a “player” in the model, and its SHAP value ($\emptyset_{j}$) represents the contribution of that feature to the model output. For a given sample, the prediction from the XGBoost model can be expressed as the sum of the baseline value (the mean value of the target variables) and the additive contributions of each feature, as shown in Equation (7):

(7) $$y=\emptyset_{0}+\sum_{j=1}^{M}\emptyset_{j}x_{j}$$

where $\emptyset_{0}$ is the base value, which is the average model output across the entire training dataset. M is the number of input features, which in our case is 4. $x_{j}$ is a binary input (where $x_{j}=1$ if feature j is present, and $x_{j}=0$ if it is absent). The SHAP values were computed using the TreeExplainer algorithm implemented in the *SHAP 0.48.0* Python package. In this paper, we choose to visualize of the SHAP results by using Beeswarm plots and dependence plots [34].

## 3. Results and Discussion

### 3.1. Accuracy and Robustness over the Tolerance Box

**Figure 7:**
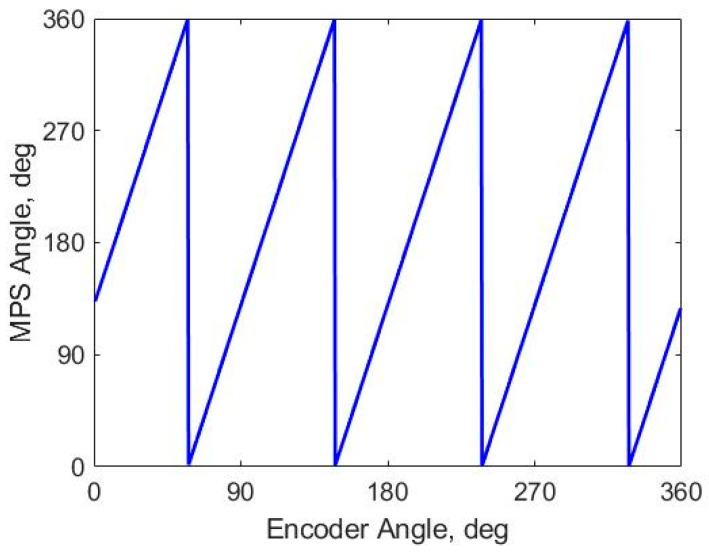
Calculated angle from MPS vs. reference encoder.

The result in Figure 7 displays the calculated electrical angle against the mechanical angle under nominal mechanical condition. The angle signal repeats four times across the 360° mechanical range, confirming the prototype is correctly configured for a motor with 4 pole-pairs (P = 4). From the figure we can also see that there is an angle offset between the encoder and the MPS angle zeros.

**Figure 8:**
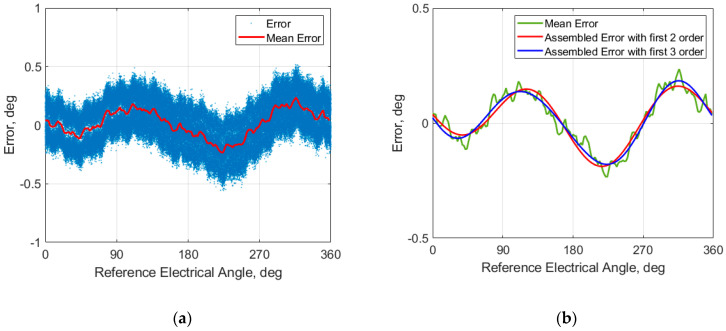
Under nominal mechanical condition: (**a**) MPS angle errors and mean errors; (**b**) comparison of mean error and assembled errors.

The corresponding electrical angle error is displayed in Figure 8a. The errors look like a cosine/sine waveform. It has two upper peaks and two lower peaks. However, the lower and upper peaks have different amplitudes. The results show that the error is dominated by 1st- and 2nd-order harmonics. In Figure 8a, the mean error is also included. The mean error was calculated by dividing the 360° into 180 bins (2° per bin) and averaging all samples within the bin. The selection of number of bins took into consideration of the data sampling rate (25 KHz) and motor speed (2000 RPM). This ensured that, at each bin, there was enough data for analysis. From the figure, we can see that the errors exhibit a relatively wide band, indicating substantial cycle-to-cycle variation. This phenomenon is mainly caused by lobe-to-lobe variation of the target (lobe shape, surface texture, and other manufacturing non-uniformities) and misalignment of the sensor and target.

The mean angle errors were used to perform Fourier decomposition to obtain the Fourier coefficients, $A_{n}$, $\varphi_{n}$, and $a_{0}$ in Equation (5). The result is summarized in Table 2. $a_{0}$ is the DC term, which is mainly determined by the $\theta_{offset}$ and carries little information about the design non-idealities. We removed the DC component before we conducted Fourier decomposition. That is why the $a_{0}$ in the table is so small and negligible. From the amplitude results listed in Table 2, it is evident that the 1st- and 2nd-order harmonics dominant the spectrum, while higher orders are much smaller. In these two dominant harmonics, the magnitude of the 2nd order is double that of the 1st order. The 2nd-order electrical harmonic error, which would arise from uncompensated amplitude mismatch. This implies that our routing strategy does affect the balance of the symmetric Rx coil. However, its impact is the about 0.135°, which is well acceptable. The appearance of the 1st-order electrical harmonic confirms that the DC offsets in Equation (4) are sensitive to airgap variation. Because the offset calibration was performed at an airgap of 3.1 mm—recommended by the IC supplier and validated in-house as the optimal calibration condition.

Figure 8b shows the comparison of the mean error over electrical cycle and reconstructed error curves based on first two harmonics and first three harmonics. The good overlap between the mean error and assembled curve by the first two harmonics confirms that the error is largely governed by the first two harmonics. Including 3rd harmonic improves approximation on the peaks but does not change the overall curve shape. This indicates the deviation from an ideal 120° electrical phase spacing in three-coil layout and lobe-to-lobe variation of the target are present but very small.

**Figure 9:**
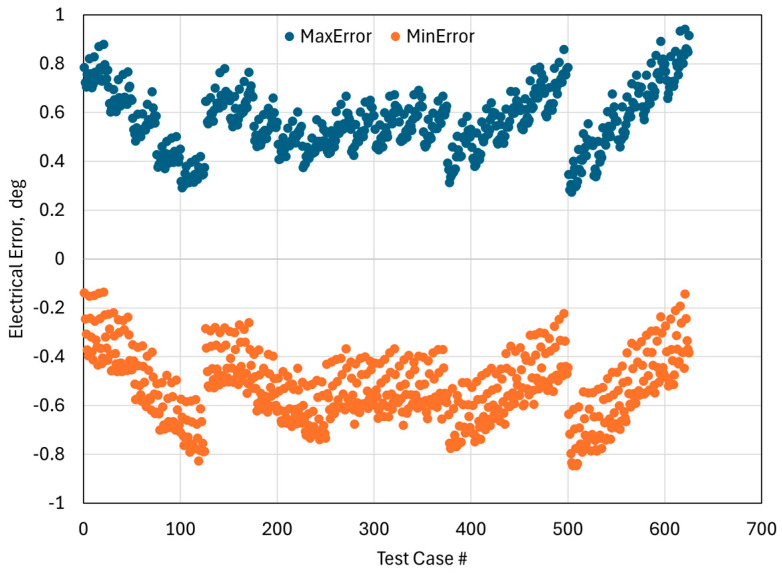
Maximum and minimum electrical angles for 625 test cases.

Figure 9 summarizes the maximum and minimum electrical angle errors across all 625 tested conditions. The Test Case # axis follows the systematic sweep of the full-factorial experimental design, with the tilt angle changing sequentially from −0.5° to 0.5°. The narrow vertical spread between the maximum and minimum values reveals that angle error remains well-bounded under mechanical perturbations. The fact that no data point exceeds the ±1 electrical-degree limit confirms the robustness of the two-layer design even in the worst-case combinations of misalignment and tilt. A pattern can be observed that increasing tilt angle generally produces a larger deviation in the measured angle (as the test case # increases, tilt angle changes from −0.5° to 0.5°). This is consistent with the expectation that tilt of the PCB or target perturbs the effective coupling area more strongly than lateral offset. Overall, the results presented in Figure 8 and Figure 9 demonstrate that the proposed limited area 2L PCB can achieve ±1 electrical-degree accuracy under all tested conditions and validated that the routing modifications does not compromise robustness.

### 3.2. XGBoost and SHAP

**Figure 10:**
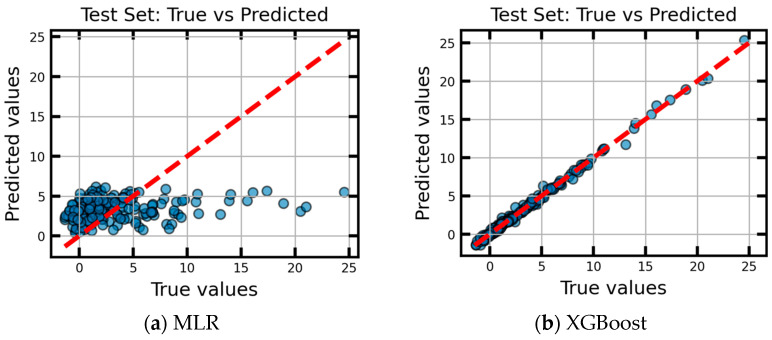
True values vs. Predicted values.

The comparison of the predictions of the final MLR and XGBoost models on the independent 30% test dataset and ground truth is shown in Figure 10. This result demonstrated XGBoost’s excellent predictive accuracy in capturing the non-linear relationships between installation geometry and sensor accuracy degradation. Table 3 summarizes both models’ performance. The coefficient of determination (R^2^) of XGBoost is 0.9951, significantly outperforming the MLR baseline of 0.0565. This demonstrates that the relationship between installation tolerances is inherently non-linear. The root mean square error (RMSE) represents the standard deviation of the residuals in the original units of the target variable. The mean absolute error (MAE) indicates the average prediction deviation. The comparison confirms that linear model fails to account for the complex geometric sensitivities. These statistics demonstrate that the XGBoost model is highly robust and reliable for accurate predicting installation-induced accuracy degradation.

**Figure 11:**
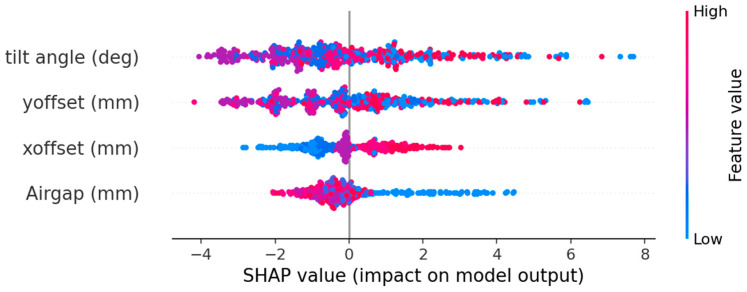
Beeswarm plot of SHAP value.

Figure 11 presents the SHAP summary (beeswarm) plot for the trained XGBoost model. Each point corresponds to one sample, with color representing the actual feature value (blue = low, red = high) and the horizontal axis showing its SHAP impact on the predicted values. Tilt angle displays the broadest spread (−4 to +8), confirming its dominant influence on sensor accuracy. The larger tilt angle leads to larger SHAP value, which as we can see from the figure that SHAP values bigger than 4 were colored red and blue. Y-offset shows the second-largest dispersion in SHAP values at approximately −3.5 to +6.5. Similarly to tilt, larger magnitude of Y-offsets, regardless of sign, lead to larger SHAP values, as shown in the figure, generally worsening accuracy. Interestingly, small Y-offsets (purple points) are associated with negative SHAP values. This behavior indicates that the modified Tx/Rx routing (shown in Figure 3) makes the system sensitive to very small Y-offsets. X-offset produces a narrower, near-symmetric SHAP distribution (−3 to +3) centered around zero. Large positive X-offsets (red points) produce positive SHAP values, while large negative X-offsets (blue points) produce negative SHAP values, and small X-offsets cluster near SHAP ≈ 0. This indicates that the Tx/Rx geometry remains symmetric in the X-direction. Airgap exhibits the small overall SHAP magnitude (−2 to +4.5), indicating a weaker contribution compared with the other factors. The lowest airgap value (blue points) tends to have positive SHAP values. In theory, a smaller airgap increases the induced voltage in the Rx coils and thus the DC offset, which, as a result, leads to the predicted accuracy degradation.

**Figure 12:**
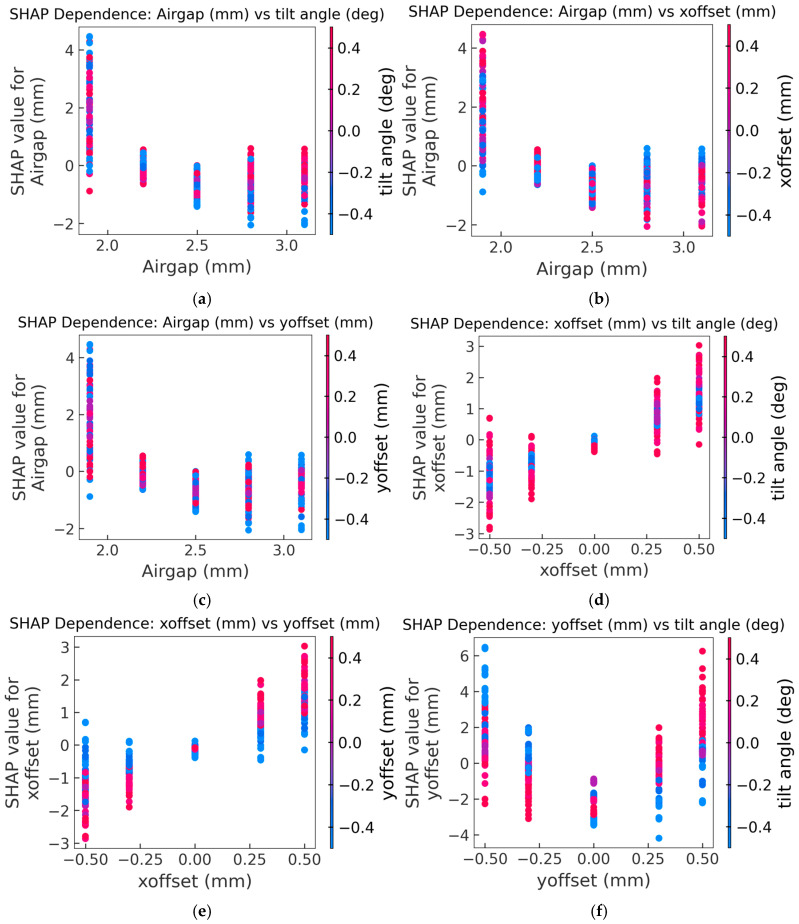
Dependence plots (**a**) airgap interacts with tilt; (**b**) airgap interact with X-offset; (**c**) airgap interacts with Y-offset; (**d**) X-offset interacts with tilt; (**e**) X-offset interacts with Y-offset; (**f**) Y-offset interacts with tilt.

The SHAP dependence plots in Figure 12 reveal how feature interactions shape performance and provide physical insight into coupled geometric sensitivities. In Figure 12a, it shows SHAP dependence of airgap with points colored by tilt angle. For small airgaps (1.9, 2.2, and 2.5 mm), the points at each level display a mixed color pattern, indicating that the SHAP contribution of airgap is largely independent of tilt and no dominant tilt effect is expressed. At the largest airgap (2.8 and 3.1 mm), however, a color separation appears: positive tilt angles (red points) are associated with higher SHAP values and negative tilts (blue points) with lower SHAP values, revealing a stronger airgap–tilt interaction in this region. Figure 12b and Figure 12c show SHAP dependence of airgap with points colored by X-offset and Y-offset, respectively. They show similar effect as Figure 12a. However, the red and blue samples in both plots remain largely intermingled at each airgap level, with no consistent vertical gradient, indicating that neither X-offset nor Y-offset influenced the specific airgap impact on sensor accuracy. Figure 12d and Figure 12e show the SHAP dependence of the X-offset feature with points colored by tilt angle and Y-offset, respectively. The X-offset dependence plot in Figure 12d shows that the sign of the SHAP contribution is mainly set by the sign of X-offset (negative offsets yield negative SHAP, positive offsets yield positive SHAP), whereas tilt angle acts as an interacting factor that changes the magnitude of this contribution rather than systematically reversing its sign. Negative Y-offset tends to pull the SHAP contribution of X-offset toward zero, while large positive Y-offset pushes it further away from zero in the direction of the X-offset sign, thereby amplifying the impact of X-misalignment on accuracy. Combined with the global SHAP ranking (Figure 11), this suggests that X-offset has a relatively small, nearly symmetric influence on the predicted target values. Its interactions with tilt and Y-offset are secondary compared with the dominant main effects of tilt and Y-offset themselves. Figure 12f shows the SHAP dependence of Y-offset with points colored by tilt angle. At Y-offset = 0 mm, SHAP values spread in a narrow range, indicating small contribution from tilt when the target is centered. As Y-offset moves away from 0 mm, the SHAP values rise sharply, especially at +0.5 and −0.5 mm, where many samples exceed +4 units, indicating that Y-offset is one of the drivers of increased target values. The color distribution indicates a pronounced interaction with tilt: positive Y-offset and positive tilt reinforce each other and jointly amplify the Y-offset contribution to error, while opposite-signed tilts partially compensate it. The result demonstrates that Y-direction misalignment—especially when combined with tilt—is one of the dominant sources of installation-induced accuracy degradation and captures the directional sensitivity introduced by the Y-aligned Tx/Rx routing modification.

**Figure 13:**
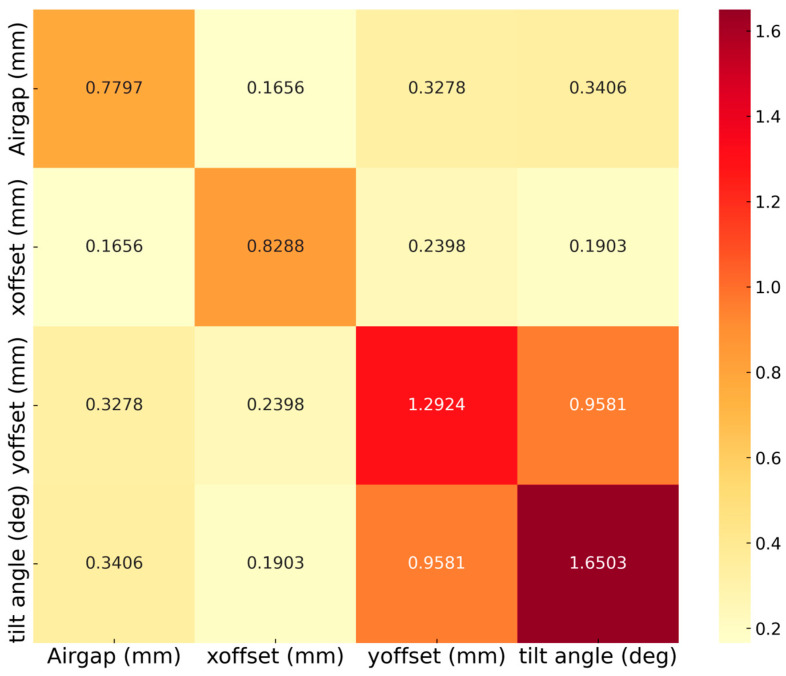
Quantitative feature interaction intensity matrix.

To quantify the complex relationship between mechanical installation tolerances, a Quantitative Feature Interaction Intensity Matrix was generated, as shown in Figure 13. This heatmap provides a numerical representation of how pairs of variables jointly influence sensor accuracy, complementing the above individual feature importance and feature interaction. The matrix reveals that the tilt and Y-offset exhibit the highest interaction intensity—0.9581, confirming that these two factors are not independent but rather reinforce each other to degrade accuracy. This aligns with the physical sensitivity introduced by modifying Tx/Rx routing. The interactions between X-offset and other variables, such as airgap (0.1656) and tilt (0.1903), are significantly smaller. These results demonstrate that while linear models like MLR might struggle with such interdependent variables, the XGBoost model effectively captures these high non-linearities.

### 3.3. Practical Implications and Generalizability of the Proposed Workflow

The present work demonstrates a general workflow that can be used to optimize and analyze inductive MPS layouts, as well as quantify installation tolerances’ impact on sensor accuracy. Importantly, this workflow is not limited to the 2L PCB configuration. It can be extended to other inductive MPS designs and layer stacks if bench data are available for the design under evaluation. The procedure consists of the following: (1) Measuring sensor accuracy under a realistic mechanical tolerance box in terms of X/Y-offsets, tilt, and airgap; (2) decomposing angle error into harmonics to identify dominant components and link them to specific design features; (3) training a regression model (here, XGBoost) as a fast surrogate of the sensor response; (4) applying SHAP analysis to rank misalignment dimensions and resolve their main effects and interactions.

In practice, the harmonic decomposition directly links to specific layout features and thus can help to optimize the sensor designs. For example, if the Fourier analysis shows a larger 2nd-order harmonic, it can point directly to Rx amplitude mismatch due to routing modification. Then, engineers can further optimize the coils routing. The SHAP results can be used by engineers to decide which factors require tight control (e.g., Y and tilt in this study) and which can be relaxed. Thus, we can provide installation suggestions to customers, focusing alignment effort on the most influential misalignment directions instead of uniformly tightening all dimensions. A practical consideration for real-world adaptation is that robust surrogate-model performance depends on collecting sufficiently installation tolerance data (including combined perturbations) to train and validate the model, which may increase experimental effort and cost. However, once established, the workflow enables rapid screening of design variants and generates actionable, data-driven guidance for both design optimization, analysis, and installation practice.

## 4. Conclusions

This paper presented and validated a compact two-layer PCB routing strategy for an inductive MPS under realistic installation tolerances. A comprehensive DOE covering 625 combinations of X/Y-offsets, tilt, and airgap (±0.5 mm in X/Y, ±0.5° tilt, 1.9–3.1 mm airgap) showed that all test cases remained within ±1 electrical degree, demonstrating that the 2L layout can satisfy typical automotive accuracy requirements. The Fourier series on the circle analysis shows that the DC offset and magnitudes mismatch of the 3 Rx signals are the dominant error contributors. An XGBoost regression model trained on test data achieved high fidelity with R^2^ = 0.9951, RMSE = 0.3105, MAE = 0.2192 compared to an MLR model with R^2^ = 0.0565, RMSE = 4.2972, MAE = 3.1009. The high predictive fidelity of XGBoost model results indicates that installation-induced accuracy degradation is inherently non-linear and not well captured by linear sensitivity baselines. SHAP analysis revealed misalignment effects. Tilt and Y-offset are the dominant contributors to accuracy degradation. X-offset and airgap exhibits a smaller influence. The SHAP interaction intensity analysis indicates a strong coupled influence between tilt and Y-offset (interaction intensity = 0.9581), demonstrating that these variables reinforce each other rather than acting independently. These results show that the proposed 2L routing achieves robust angle accuracy across realistic misalignment ranges and that the workflow offers a general, quantitative approach for analyzing inductive MPS layouts and installation tolerances in future designs.

Future work will focus on identifying quantitative dimensional thresholds for both 2-layer and 4-layer PCB configurations. Establishing these boundaries will enable engineers to make informed cost–performance trade-offs and to select the most appropriate layer configuration for a given application, thereby guiding the deployment of next-generation, low-cost inductive MPSs with predictable installation robustness.

## Figures and Tables

**Table 1:** DOE summary.

Factor	Levels	Motor Speed (RPM)
Airgap (mm)	[1.9, 2.2, 2.5, 2.8, 3.1]	2000
X-offset (mm)	[−0.5, −0.3, 0, 0.3, 0.5]	2000
Y-offset (mm)	[−0.5, −0.3, 0, 0.3, 0.5]	2000
Tilt θ (deg)	[−0.5, −0.3, 0, 0.3, 0.5]	2000

**Table 2:** Harmonic coefficients.

Order	$\mathrm{Amplitude}\;A_{n}$ (deg)	$\mathrm{Phase}\;\varphi_{n}$ (rad)
0 ($a_{0}$)	6.5534 × 10^−18^	0
1	0.0686	−0.5592
2	0.1350	1.8352
3	0.0234	2.2289
4	0.0200	−0.9133
5	0.0032	−0.4544
6	0.0158	−1.3024
7	0.0025	1.5137
8	0.0020	−0.1020
9	0.0014	−0.5425
10	8.7534 × 10^−4^	−3.0656

**Table 3:** Performance comparison between MLR and XGBoost.

Metric	MLR (Baseline)	XGBoost
R^2^ score	0.0565	0.9951
RMSE	4.2972	0.3105
MAE	3.1009	0.2192

## Data Availability

Processed dataset for ML training (features and targets) is provided in Supplementary Materials; raw unprocessed bench data remains confidential.

## Supplementary Materials

The following supporting information can be downloaded at: https://www.mdpi.com/article/10.3390/s26030879/s1.

## Glossary

Summary of key symbols used in the paper:

**Symbol**	**Unit**	**Definition**
$\theta_{mps}$	deg	Calculated electrical angle from inductive MPS
$\theta_{ref}$	deg	Reference electrical angle converted from encoder angle
$\theta_{offset}$	deg	Electrical angle offset between MPS and encoder
$\varphi_{i}$	deg	Encoder measurements
N	-	Number of pole-pairs
U, V, W	mV	Rx coils induced voltage
$A_{u}$$,\;A_{v}$$,\;A_{w}$	mV	Harmonic coefficients of the 3 Rx signals
$c_{u}$$,\;c_{v}$$,\;c_{w}$	mV	DC offset
$A_{n}$	deg	Magnitude of nth harmonics
$a_{0}$	deg	DC term in Fourier Error decomposition
$k_{i}$	-	Regression coefficient
$x_{i}$	-	Installation tolerance
$\emptyset_{j}$	-	SHAP value
y	-	Target variable
